# Effects of Superfine Grinding on Physicochemical Properties and In Vitro Starch Digestibility of Red Rice Powder

**DOI:** 10.3390/molecules31203622

**Published:** 2026-10-11

**Authors:** Xiaojuan Guo, Liang Shuai

**Affiliations:** 1 College of Chemistry and Food Science, Nanchang Normal University, Nanchang 330023, China; 2 Jiangxi Provincial Research Center for National Nutrition Health Education, Nanchang 330023, China

**Keywords:** red rice, superfine grind, physicochemical properties, starch digestibility

## Abstract

Pigmented red rice is a promising functional whole grain, but conventional milling cannot fully release its intracellular bioactives due to dense bran cell walls. Superfine grinding can effectively destroy compact tissue structures and improve the processing characteristics of cereal powders. In this study, red rice powders (RRPs) with different particle sizes were prepared by superfine grinding, and their physicochemical properties, micromorphology, crystalline structure, total phenolic content (TPC), pasting behaviors, and in vitro starch digestibility were tested by particle size analysis, colorimetric measurement, SEM, XRD, FT-IR, the Folin–Ciocalteu method, RVA, and an in vitro simulated digestion model. The results show that superfine grinding caused severe matrix destruction, reducing the particle size (D90) by 83.5%, and remarkably increasing the specific surface area. Furthermore, superfine grinding improved color brightness, decreased powder flowability, and reduced starch crystallinity, while no new functional groups were generated. Additionally, the TPC and water/oil-holding capacity were effectively enhanced. The pasting properties and in vitro starch digestibility of RRP were modified by superfine grinding. This study provides basic data on RRP properties and recommends the selection of proper grinding intensity in order to obtain powder for diverse food applications, such as functional beverages and bakery products.

## 1. Introduction

With the rise in global public health consciousness and increasing consumer trends toward balanced dietary structures, whole-grain foods rich in natural phytochemicals have attracted scientists’ attention in recent years [1]. Compared with refined grains that undergo polishing procedures, whole-grain products retain complete bran and germ fractions, which are regarded as concentrated reservoirs of diverse health-promoting components. Pigmented cereals are enriched in bran pigments and bioactive substances, conferring greater exploitation value compared with refined cereals [2,3]. Red rice (*Oryza sativa* L.) is characterized by pigmented bran containing substantial bioactive substances [4,5], making it a promising basic ingredient in diverse food products, such as products targeted towards the elderly [6], cookies [7], gluten-free food [8], etc.

Red rice consists of a dense and rigid outer bran layer, in which intact cell walls act as physical barriers. Conventional crushing merely produces coarse fragments, leaving intracellular bioactive compounds confined within the cell walls, leading to a lower efficiency of nutrient utilization [9]. Accordingly, eco-friendly physical modification strategies are urgently required to disrupt these structural barriers and improve the utilization efficiency of bioactive components in red rice.

Recent advances in food powder processing technology confirm that superfine grinding is an emerging green and highly effective physical modification method for enhancing the quality and industrial utility of cereal-based ingredients [10,11]. Particle micronization induced by superfine grinding significantly changes the microscopic morphology and structural characteristics of raw grain materials [12]. This mild physical modification effectively disrupts compact cell wall networks, facilitating the release of bound phenolic compounds [13,14]. Meanwhile, pronounced particle size reduction improves mouthfeel uniformity and facilitates flavor release in final food products [14,15]. However, excessive superfine grinding can cause undesired effects, including the degradation of heat-sensitive nutrients, accelerated lipid oxidation, powder agglomeration and poor flowability, as well as shortened shelf life. As red rice contains lipid-rich bran and oxidation-prone phenolic substances, such negative impacts deserve special attention. Therefore, an optimal range of particle sizes should be determined based on the properties of raw materials and the requirements of targeted food applications.

In terms of processing mechanisms, the technical parameters and grinding modes of superfine grinding largely determine the mechanical force patterns, heat generation, particle collision intensity and degree of starch damage during pulverization, which further modulate the macroscopic physicochemical characteristics, microscopic structural features and in vitro starch digestibility of the resulting grain powders. Grain powders with distinct particle sizes usually exhibit markedly different physicochemical properties, structural characteristics and processing adaptability, making them suitable for various food applications [16,17,18]. Numerous studies have investigated the superfine grinding of brown rice, wheat and other cereals. However, the conclusions derived from these cereal materials cannot be directly extrapolated to red rice systems. On the one hand, pigmented red rice exhibits unique material characteristics, such as the grain morphology, the compactness of the bran layer, starch composition, etc. On the other hand, the effects of superfine grinding are strongly dependent on grinding modes and particle-size ranges. Even for similar cereal raw materials, variations in pulverization equipment and resulting particle-size gradients can induce remarkably divergent physicochemical, structural and digestive properties in the powder. For this reason, the optimal processing parameters established for other pigmented cereals are not fully applicable to red rice. Nevertheless, the systematic experimental evidence on how different superfine grinding intensities affect the processing adaptability of red rice powder (RRP) remains insufficient.

In this study, red rice was subjected to superfine grinding to obtain samples with different particle sizes. Multiple indicators including particle size, color parameters, tap density, repose angle, slide angle, SEM morphology, XRD patterns, FT-IR spectra, water-holding capacity, oil-holding capacity, TPC, pasting properties and in vitro starch digestibility were measured to systematically characterize the changes in RRP properties upon particle size reduction. This study provides comprehensive data on the property evolution of superfine ground red rice powder, and offers an experimental basis for selecting appropriate superfine grinding intensity to produce red rice powder for diverse food applications.

## 2. Results and Discussion

### 2.1. Particle Size Distribution

**Figure 1:**
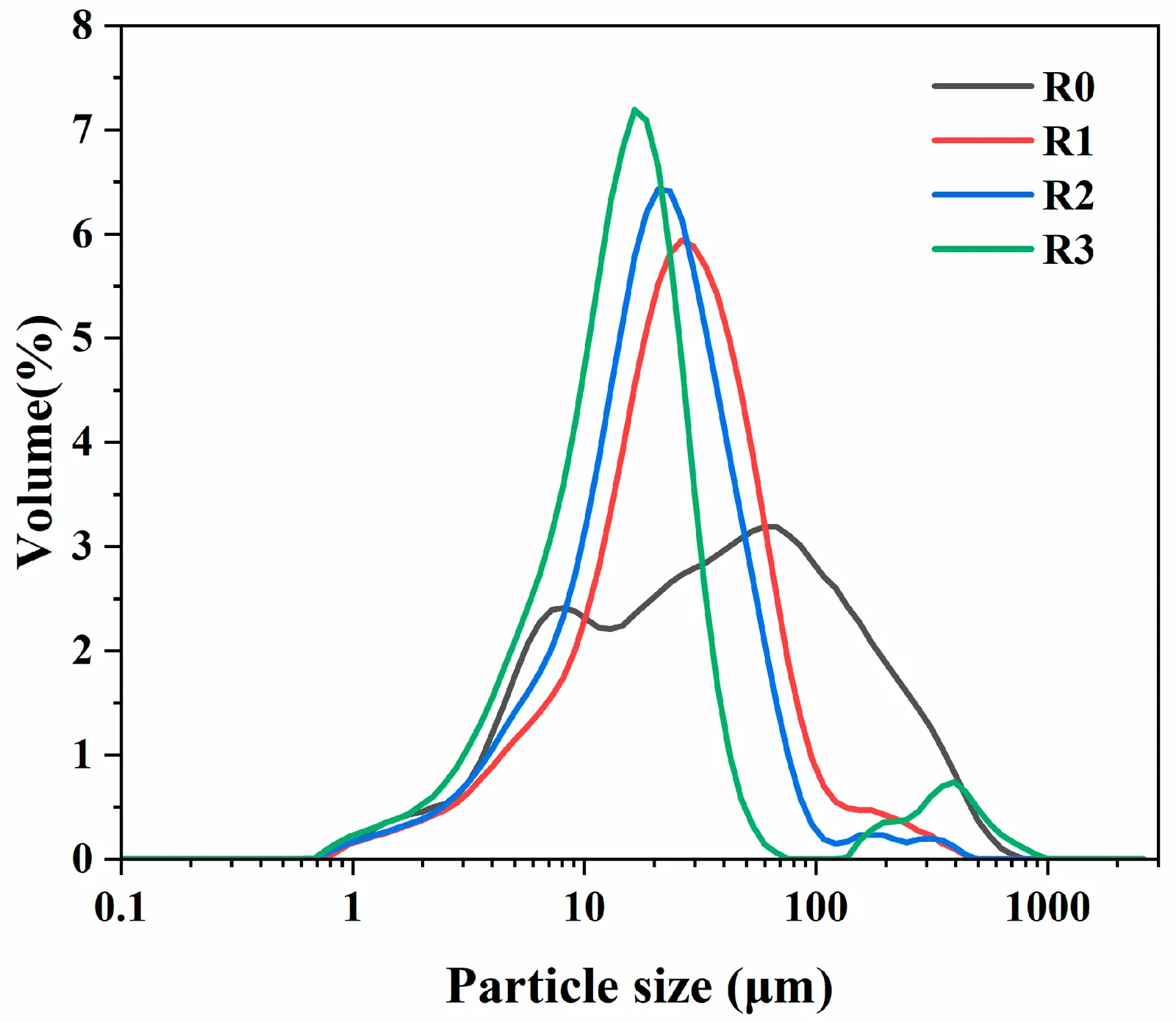
Effects of superfine grinding on particle distribution of RRP.

The particle size indices of RRP are shown in Table 1. The RRP samples exhibited distinct particle sizes. The D(50) of R3 was the lowest (14.0 μm), which was lower than those of R0 (35.6 μm), R1 (23.8 μm) and R2 (19.1 μm). The D(10) and D(90) values showed the same trend. Meanwhile, the specific surface area of R3 (255.8 m^2^/kg) was higher than that of the other three groups, indicating that intensified superfine grinding treatment could strengthen the pulverization effect of RRP. Figure 1 shows the effects of different superfine grinding intensities on the particle size distribution of RRP. The coarsely ground group R0 presented a bimodal distribution, containing a primary peak within 1–100 μm and a secondary broad peak at 100–1000 μm. As the superfine grinding intensity increased from R0 to R3, apparent variations in peak shape and position were observed. The secondary peak located at 100–1000 μm continuously declined in volume proportion and shifted toward smaller particle sizes, accompanied by an increased peak height and a narrowed distribution width. Accordingly, a smaller particle size and larger specific surface area usually imply the greater exposure of starch and bioactive components, which may facilitate interactions with digestive enzymes or food matrices.

### 2.2. Color Difference

As presented in Table 2, distinct differences in color parameters were observed among RRP samples with different particle sizes. The superfine grinding treatment altered the L*, a* and b* values of RRP. The L* value representing lightness showed an overall upward trend. R3 exhibited the highest L* value (61.11), which was higher than those of R0, R1 and R2. However, the a* and b* values decreased with the decreasing particle size of RRP. These results indicate that superfine grinding made RRP brighter and less red and yellow. This may be due to the increased specific surface area after particle refinement, which strengthened light scattering and thus improved lightness [19,20]. This characteristic may help improve the sensory acceptability of RRP and broaden its application prospects in the food industry.

### 2.3. Flow Property

**Figure 2:**
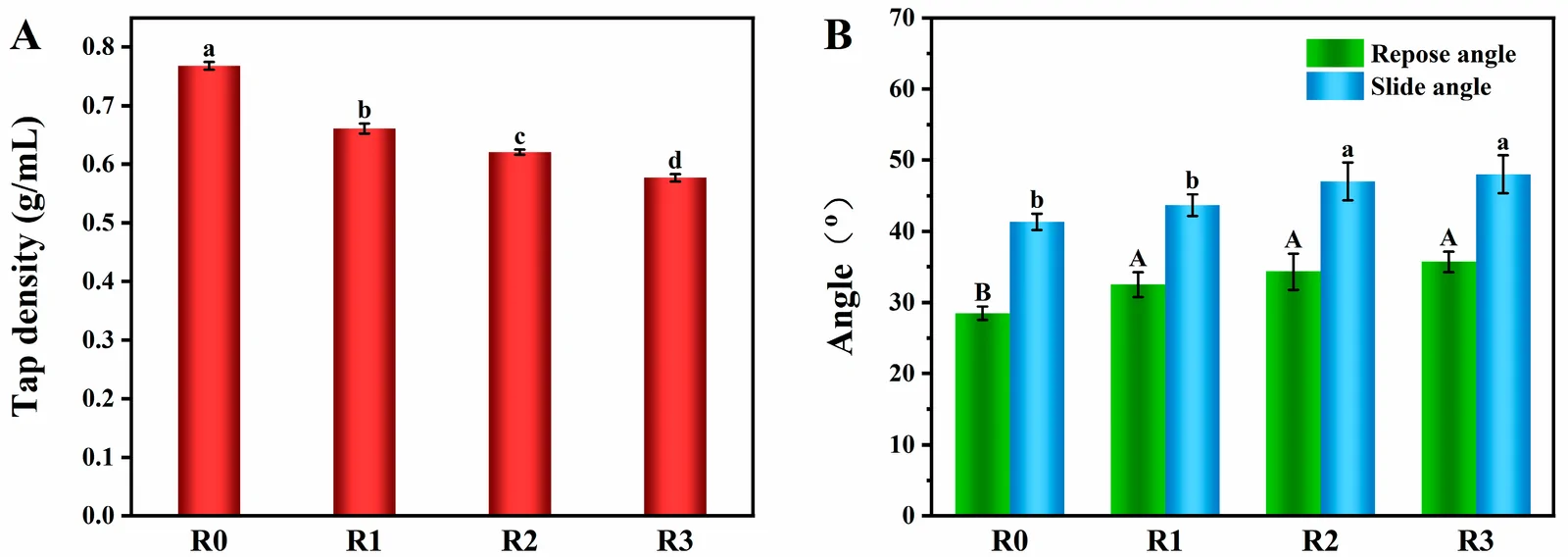
Effects of superfine grinding on tap density (**A**), repose angle and slide angle (**B**) of RRP. Different letters above bars of the same color are used to group samples with numerically different values.

Flowability is a critical quality attribute used in powder selection, characterized by tap density, repose angle and slide angle. As shown in Figure 2A, the tap density of RRP decreased after superfine grinding. The results of Zhang et al. [21] also show that the tapped density of *Tremella fuciformis* powder decreased following superfine grinding treatment. In addition, the repose angle and slide angle of RRP increased after superfine grinding. These larger angles correspond to reduced flowability, which is consistent with the results of Li et al. [22]. It can be inferred that superfine grinding reduced the spacing of adjacent particles, which amplified the intermolecular electrostatic force and van der Waals force and further impeded particle movement [23].

### 2.4. Scanning Electron Microscope Analysis

**Figure 3:**
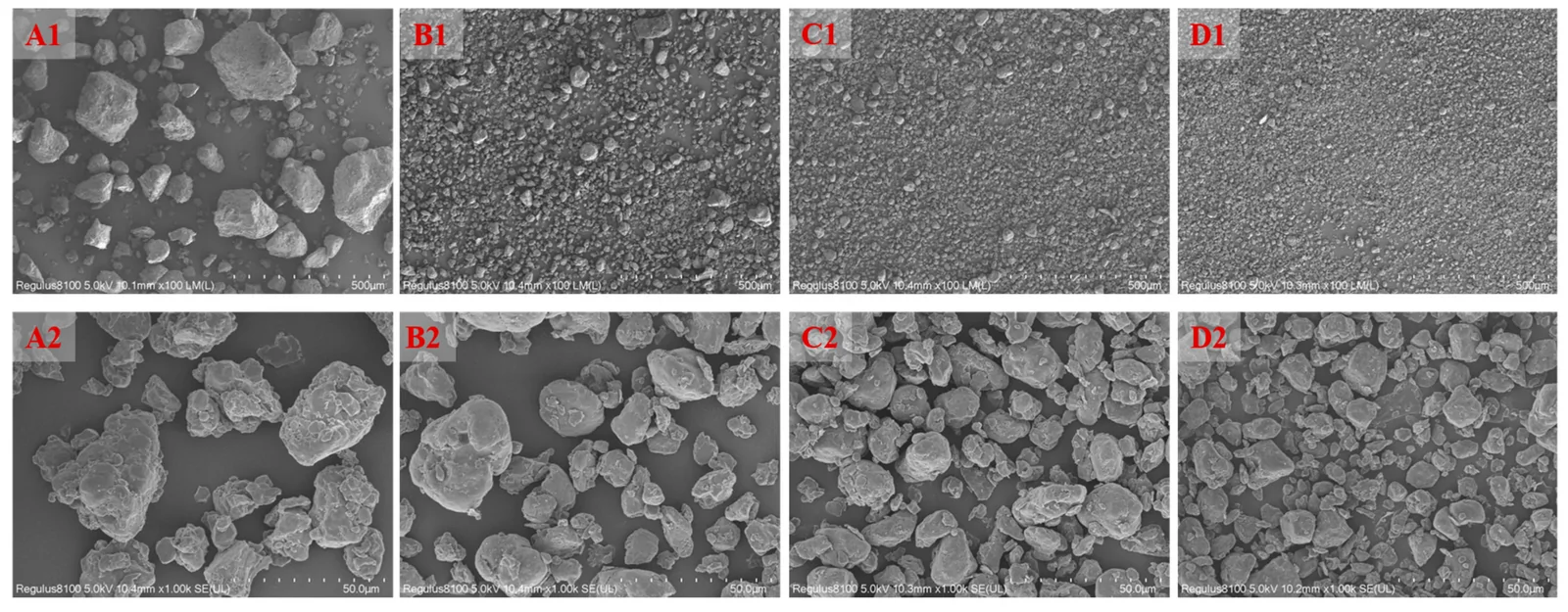
Effects of superfine grinding on microstructures of RRP. (**A1**–**D1**) Magnification 100× of R0–R3, respectively; (**A2**–**D2**) magnification 1000× of R0–R3, respectively.

The effects of superfine grinding on the microstructure of RRP are shown in Figure 3. Compared with the coarse powder R0, the granules of R1, R2 and R3 (Figure 3(A1–D1)) were severely destroyed, which corresponds closely with the results for particle size illustrated in Table 1 and Figure 1. Additionally, the particle morphology of RRP was altered (Figure 3(A2–D2)). Specifically, superfine grinding endowed RRP with well-regulated particle geometries, an elevated fraction of spherical particles and smoother outer surfaces. Overall, superfine grinding changes the morphological characteristics of powder particles, and such structural modifications may further affect the powders’ physicochemical properties and functional behaviors [24].

### 2.5. XRD

**Figure 4:**
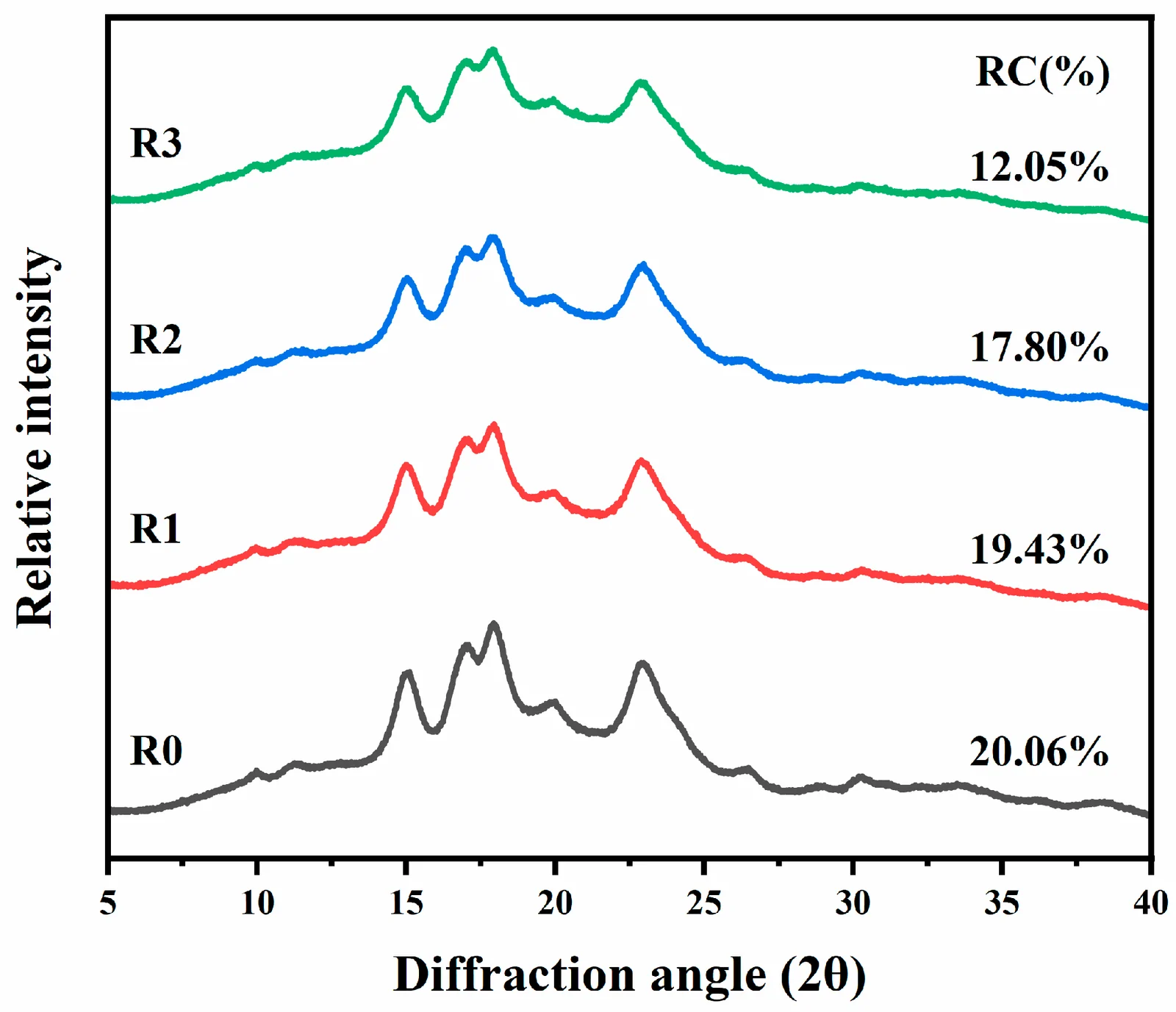
X-ray diffraction patterns and relative crystallinity of RRP.

X-ray diffraction analysis was performed to investigate the changes in the crystalline structure of RRP treated by superfine grinding, and the results are presented in Figure 4. As can be observed in the XRD patterns, all samples displayed the standard A-type crystalline configuration of cereal starches, which contained two isolated peaks at 15° and 23°, as well as an unresolved doublet peak at 17° and 18°. However, the crystalline peaks weakened with intensified superfine grinding treatment. Consistently, the relative crystallinity (RC) decreased from 20.06% (R0) to 12.05% (R3). The reduced crystalline peaks and crystallinity indicate that the starch in RRP was destroyed after superfine grinding treatment [25]. In previous studies, Luo et al. [12] and Liu et al. [11] reported analogous findings, which demonstrated that superfine grinding treatment weakened crystalline diffraction peaks and reduced the crystallinity of cereal materials.

### 2.6. FT-IR

**Figure 5:**
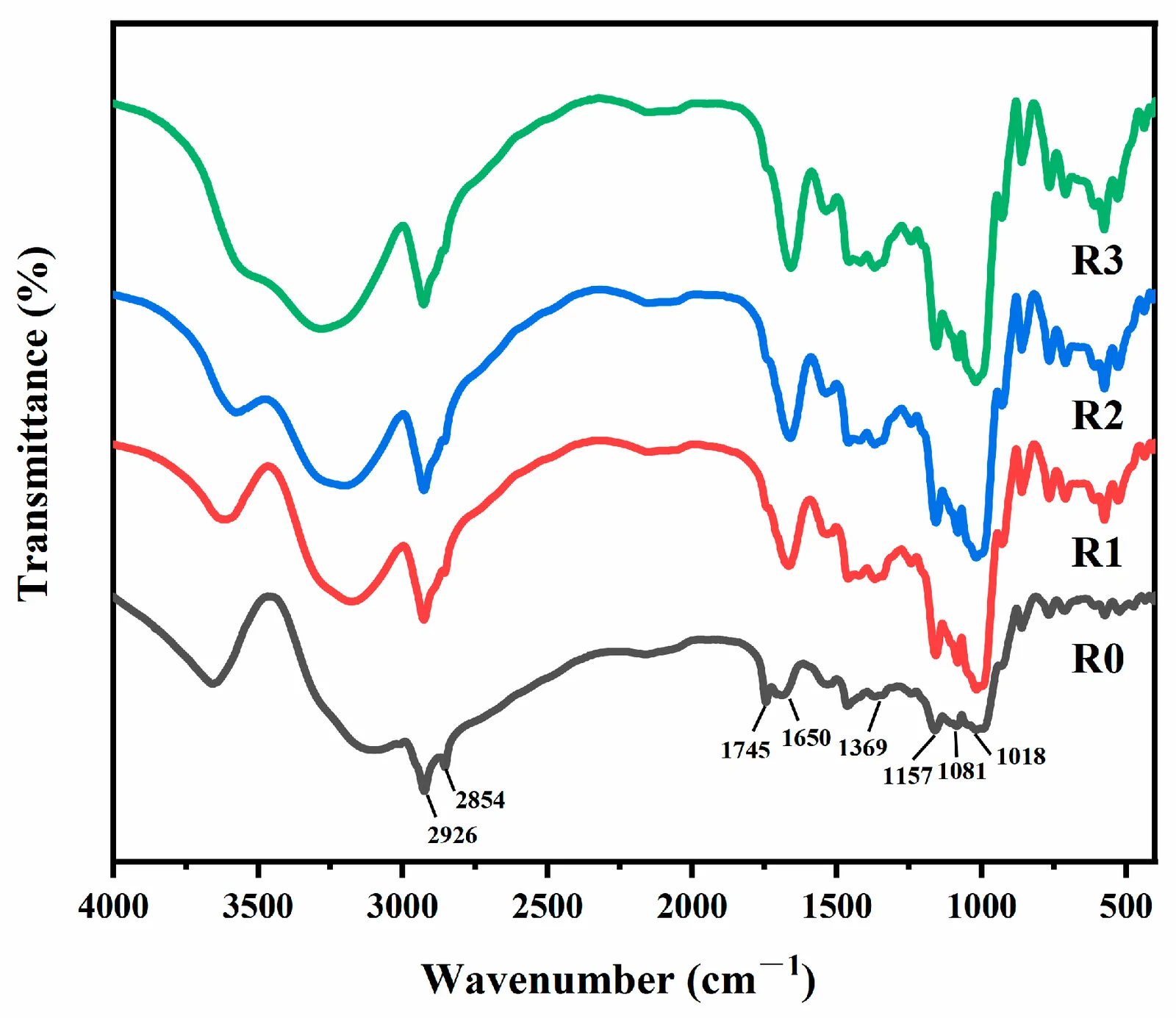
FT-IR spectrum of RRP.

The FT-IR results of RRP are shown in Figure 5. According to the transmittance of the absorption peak, all samples showed similar spectra with absorption peaks near 4000 cm^−1^~3000 cm^−1^, 2926 cm^−1^, 2854 cm^−1^, 1745 cm^−1^, 1650 cm^−1^, 1369 cm^−1^, 1157 cm^−1^, 1081 cm^−1^ and 1018 cm^−1^. The absorption peak located at 4000 cm^−1^~3000 cm^−1^ corresponds to the stretching vibrations of intra- and intermolecular O–H [26]. The peaks at 2926 cm^−1^ and 2854 cm^−1^ originated from the –CH_2_ stretching vibration from polysaccharide methylene groups [27,28]. The peaks at 1745 cm^−1^ and 1650 cm^−1^ are characteristic C=O vibration bands assigned to lignin or hemicellulose components [29]. The absorption peak at 1369 cm^−1^ is assigned to the bending vibrational modes of C–H groups [30]. The characteristic peaks located at 1018, 1081 and 1157 cm^−1^ correspond to stretching vibrations of C–O bonds [26]. No obvious new absorption peaks were observed, suggesting that superfine grinding treatment mainly reduced the particle size of RRP without obviously generating new functional groups or markedly altering the primary chemical structure of red rice. This phenomenon is consistent with the results of Yi et al. [31].

### 2.7. TPC

**Figure 6:**
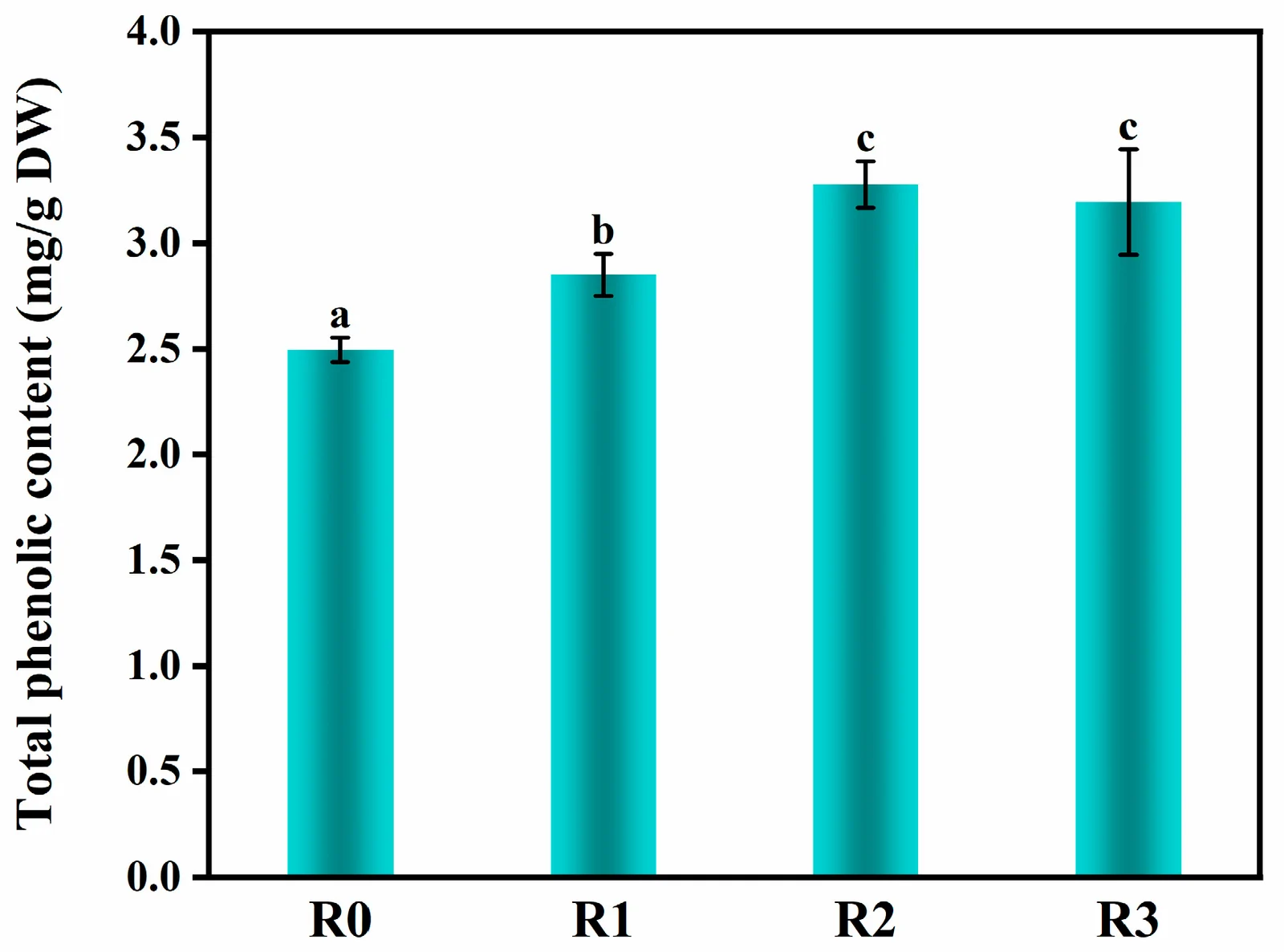
Effects of superfine grinding on TPC of RRP. Different letters are used to group samples with numerically different values.

The TPC of RRP increased after superfine grinding (Figure 6 and Table S1). The coarsely ground group R0 exhibited the lowest polyphenol content. After superfine pulverization, the TPC of R1 increased. Further grinding led to a continuous increase in TPC, and R2 achieved the maximum value. The TPC of R3 was similar to that of R2. The increased TPC could be attributed to the mechanical disruption of compact bran cell walls during superfine grinding. Particle size reduction destroys intact cell structures, releasing the bound phenolic compounds embedded in cell wall matrices [9]. However, extending the grinding treatment further did not continuously increase the detectable phenolic compounds. This phenomenon suggests that cell wall fragmentation nearly reached saturation in the R2 group, and additional pulverization failed to release more bound polyphenols.

### 2.8. Water-Holding Capacity (WHC) and Oil-Holding Capacity (OHC)

**Figure 7:**
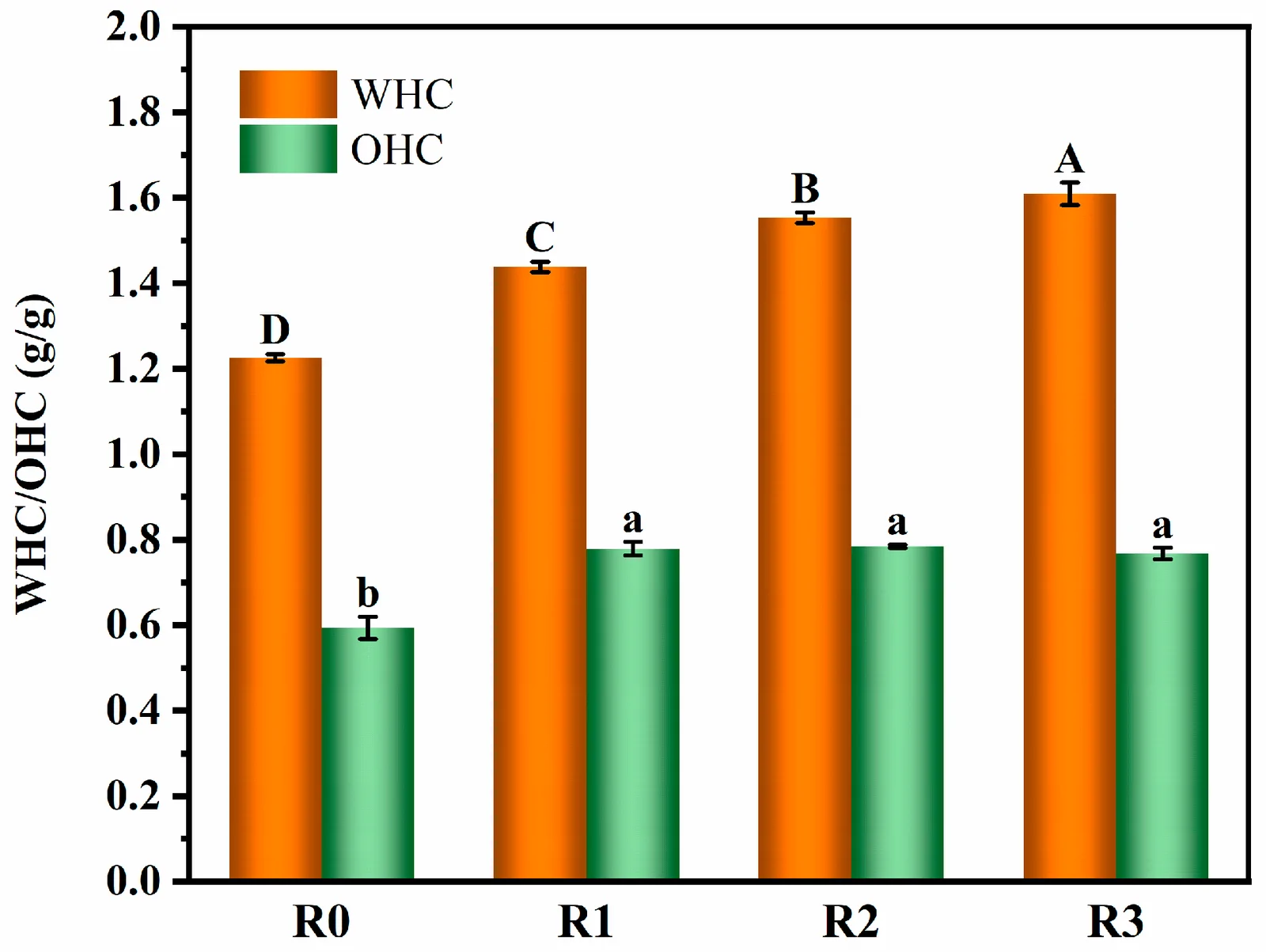
Effects of superfine grinding on WHC and OHC of RRP. Different letters above bars of the same color are used to group samples with numerically different values.

As shown in Figure 7 and Table S1, the WHC of RRP gradually increased with increasing grinding intensity, rising from 1.26 g/g to 1.56 g/g. The results obtained in this study are consistent with the findings reported by Zhu et al. [32]. The OHC of RRP also increased after superfine grinding treatment. Based on the particle size and SEM results, the above variations in WHC and OHC can be explained by the following mechanisms. Superfine grinding treatment increased the specific surface area of RRP, thereby exposing additional hydrophilic and lipophilic active sites. Mechanical crushing destroyed the intrinsic cell wall architecture of RRP, liberating intracellular starch, proteins, dietary fiber and other bioactive constituents. These macromolecules are rich in hydrophilic moieties capable of interacting with water molecules through hydrogen bonding. Additionally, the exposed nonpolar functional groups facilitate the stronger adsorption and entrapment of lipid molecules.

### 2.9. Pasting Properties of RRP

**Figure 8:**
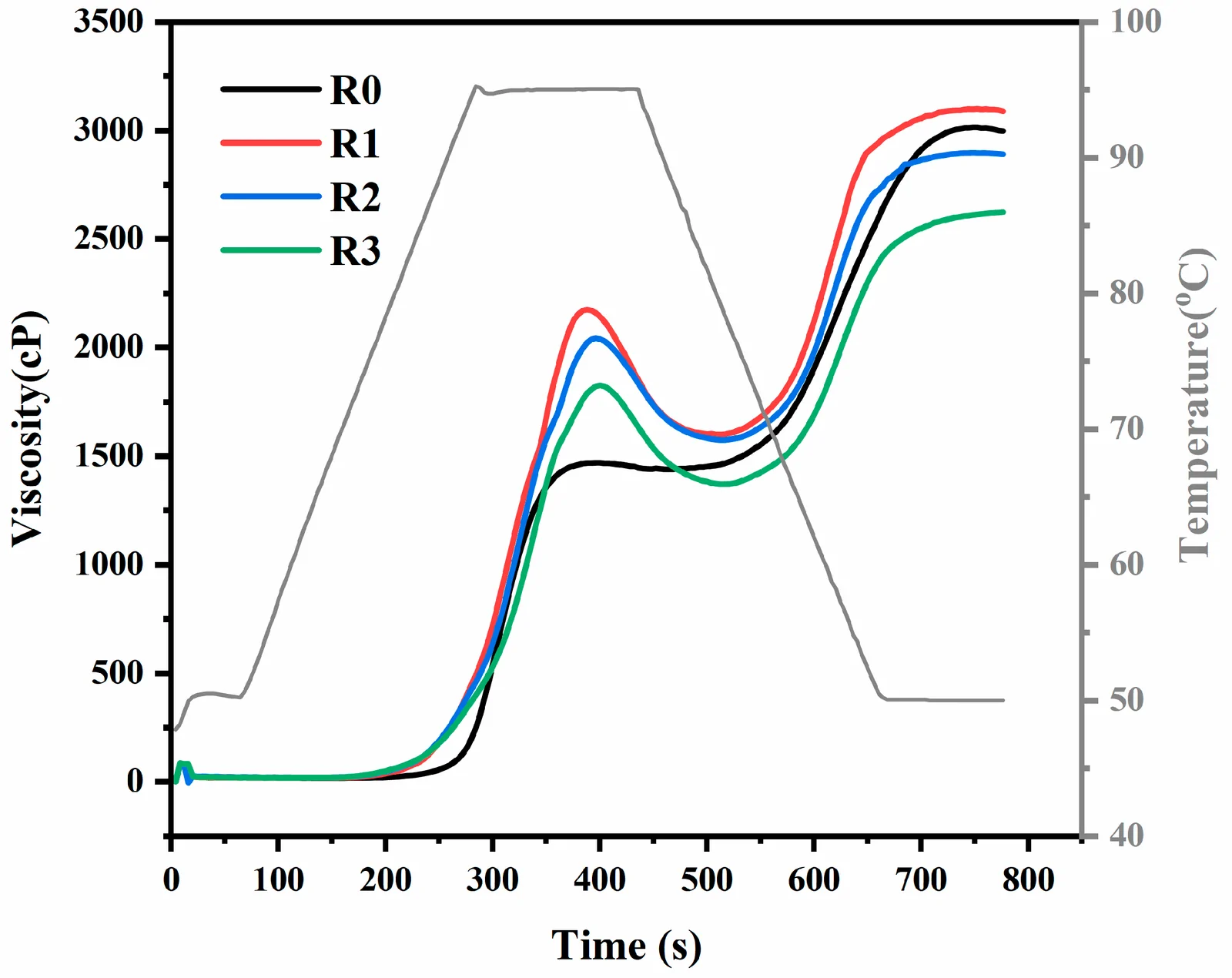
Effects of superfine grinding on the RVA profile of RRP.

Pasting properties exert a critical influence on the physicochemical quality of RRP as well as its derived finished products [25]. Accordingly, the pasting profiles of RRP were systematically characterized in this work. RVA was employed to measure the pasting parameters of RRP, including pasting temperature (PT), peak viscosity (PV), trough viscosity (TV), final viscosity (FV), breakdown (BD) and setback (SB). All measured data are listed in Table 3, and the typical pasting profiles are presented in Figure 8. The pasting properties of RRP were affected by superfine grinding treatment. Consistent with the present findings, Guan et al. [33] also reported that superfine grinding significantly influenced the pasting behaviors of cereal powders. Compared with coarse powder R0, the lightly pulverized sample R1 displayed increases in PV, TV and FV. The partial destruction of the compact bran layer under mild grinding eliminated the physical barrier for water penetration, enabling starch granules to fully absorb water and swell during gelatinization. Accordingly, R1 showed the highest viscosity and a higher BD value, indicating a weakened structural resistance of starch granules to thermal shear. With the elevatation of grinding severity from R1 to R3, a gradual downtrend was detected for PV, TV and FV. This may be because severe mechanical collision and shear force caused the fragmentation of starch granules, leading to continuous declines in various viscosity parameters. Notably, this gradual deterioration in pasting viscosity corresponded well with the reduced starch crystallinity determined by XRD analysis. Specifically, the consistent drop in SB demonstrated a progressive improvement in anti-retrogradation performance, and R1, R2 and R3 retained lower PT than R0, suggesting that superfine grinding effectively promoted starch gelatinization. The results suggest that mild superfine grinding may be a promising strategy for improving the viscosity of food matrices, while heavy superfine grinding appeared to suppress starch retrogradation. The raw coarse powder, in contrast, exhibited poor gelatinization performance and a stronger retrogradation tendency.

### 2.10. In Vitro Starch Digestibility

**Figure 9:**
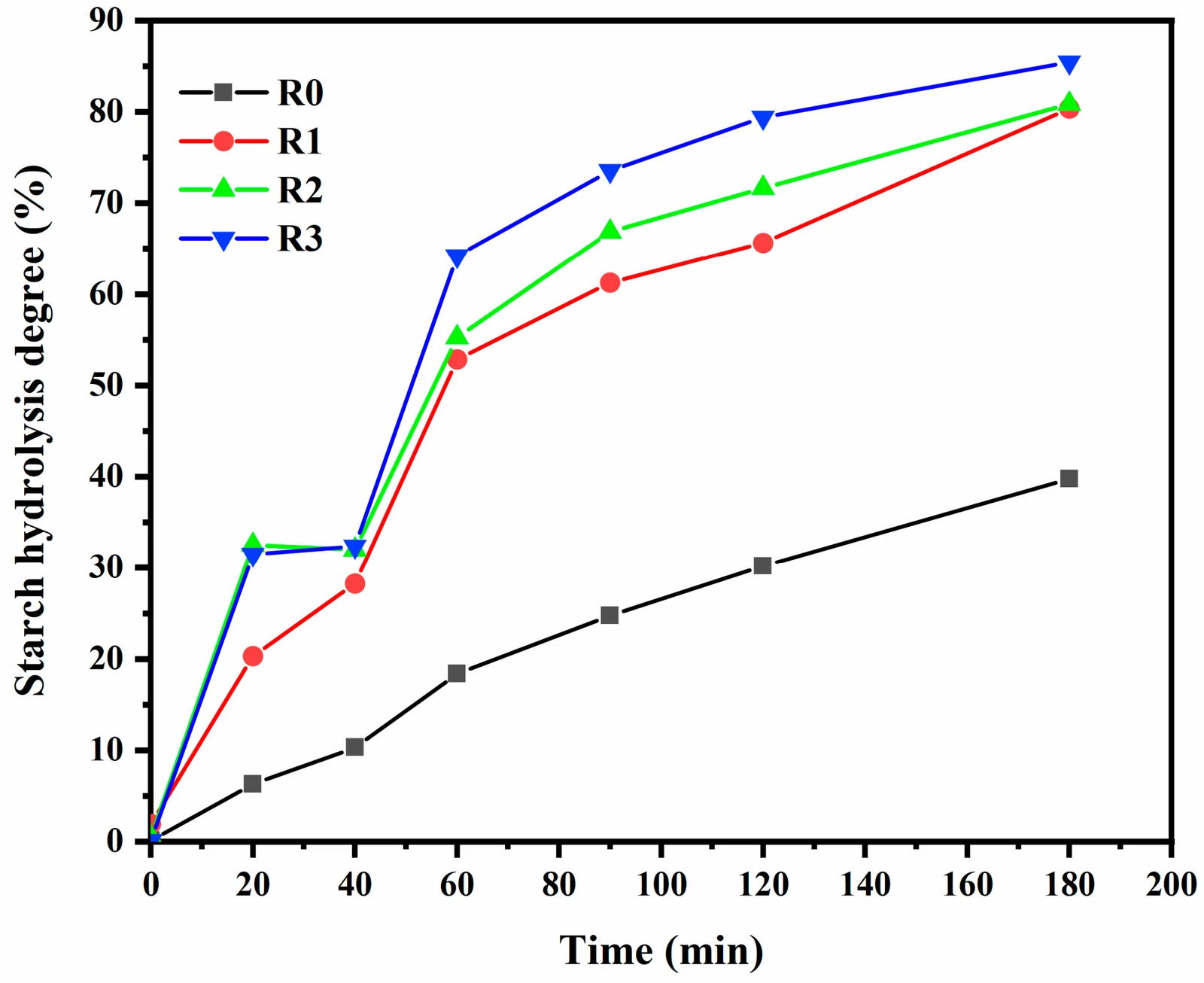
Effects of superfine grinding on in vitro hydrolysis profiles of starch in RRP.

Figure 9 and Table S1 illustrate the variation in the degree of starch hydrolysis of the samples during in vitro simulated digestion as a function of digestion time. The degree of starch hydrolysis of all samples continuously increased with increasing digestion time, exhibiting a typical enzymatic digestion kinetic characteristic of rapid hydrolysis at the initial stage and decelerated reaction rate in the later stage. The overall digestibility of each group followed the order R3 > R2 > R1 > R0. For the coarsely ground group R0, the intact cell wall matrix of the powder exhibited a compact structure. Such initial structural characteristics limit the accessibility of starch to amylase in subsequent hydrolysis, resulting in the lowest hydrolysis rate over the whole digestion period. In contrast, superfine grinding disrupted the native microstructure of R1, R2 and R3 samples, increased their specific surface area, and provided more available binding sites for amylase after cooking, which substantially raised the digestion rate and final hydrolysis extent [34]. In summary, superfine grinding treatment can enhance the in vitro digestibility of starch, and the starch hydrolysis capacity rose with the increase in grinding intensity.

## 3. Materials and Methods

### 3.1. Materials

Red rice was purchased from Jinggangshan Ganxin Planting and Breeding Professional Cooperative (Jinggangshan, China). The porcine pancreas α-amylase (Type VI-B, A3176, 13 U/mg) and amyloglucosidase (A7095, 260 U/mL) were bought from Sigma-Aldrich (St. Louis, MO, USA). The D-glucose Assay Kit (GOPOD Format K-GLUK) and Total Starch Assay Kit (K-TSTA) were obtained from Megazyme International Ltd. (Wicklow, Ireland). Unless otherwise specified, all other chemical reagents were of analytical grade.

### 3.2. Preparation of RRP

RRP was prepared using a superfine pulverizer (CWF-300S, Kangyuan Pharmaceutical Machinery Co., Ltd., Rui’an, China). The speed of the rotor was set at 5000 rpm, and the feeding frequency of the screw feeder was set at 15 Hz. An external circulating chiller was used for chamber cooling, with the cooling water temperature maintained at 5 °C, and the outlet powder temperature was kept below 40 °C during grinding. The particle size of the resulting powder was controlled by adjusting the fineness regulation dial mounted on the grinding chamber; turning the handle toward the fine end narrows the gap between the impeller and the grinding chamber wall, thereby yielding finer particles. Fractions R0, R1, R2, and R3 were obtained by setting the dial to positions 1, 2, 3, and 4, respectively, from the initial (coarsest) position.

### 3.3. Particle Size Measurement

The mean diameter of the particles in the RRP was determined using a laser diffraction particle size analyzer (Bettersize2600, Dandong Bettersize Instruments Ltd., Dandong, China) according to a previously reported method [35]. The mean diameter was evaluated based on volume weighted mean diameter, D[4,3], and surface-weighted mean diameter, D[3,2]. Cumulative percentiles D10, D50 and D90 were also determined, indicating that 10%, 50% and 90% of the particles fell below the specified diameter, respectively. Additionally, the specific surface area was determined.

### 3.4. Color Analysis

The color parameters of RRP were analyzed by use of a chromometer (CM-23d, Konica Minolta, INC., Tokyo, Japan). The samples were analyzed in clean glass dishes. A white plate was used as a standard to calibrate the instrument. The lightness (L*), redness/greenness (a*) and yellowness/ blueness (b*) were recorded.

### 3.5. Tap Density

RRP was poured into a pre-weighed graduated cylinder up to the 10 mL mark. The cylinder was tapped repeatedly until the volume of the powder remained constant. The final volume was recorded, and the total mass of the cylinder together with the powder was weighed. The tap density (ρ) was calculated using the following equation.

*ρ* = (*m*1 − *m*2)/*V*

Here, m1 and m2 represent the total mass of the cylinder together with the powder and cylinder (g), respectively. V is the volume of the samples after tapping (mL).

### 3.6. Repose Angle and Slide Angle

The repose angle (β) of RRP was measured according to a previously described method [21]. During testing, a vertical glass funnel was positioned over the flat test bench. Powder was slowly added into the funnel to build up a conical stack. The cone’s height and bottom radius were then documented, and β was computed via the following equation.

*β* = *arctan*(*H*/*R*)

Here, H is the height of the powder cone, and R represents the base radius of the powder cone of the sample.

The slide angle of RRP was determined by the inclined plate method. An accurately weighed amount of RRP was spread evenly on a smooth glass plate with gentle shaking. One side of the glass plate was slowly lifted upward until the RRP started sliding down. The included angle between the glass plate and the horizontal plane was recorded as the slide angle.

### 3.7. Scanning Electron Microscope (SEM)

The samples were fixed on a metal sample holder using double-sided conductive adhesive and sprayed with gold. The micrographs of RRP were captured using SEM (Regulus 8100, Hitachi, Tokyo, Japan) at 100× and 1000× magnification with an acceleration voltage of 5 kV.

### 3.8. X-Ray Diffraction (XRD) Analysis

An X-ray diffractometer (SmartLab SE, Rigaku, Tokyo, Japan) was adopted to record the diffraction profiles of RRP. The integral calculation of amorphous and crystalline regions was conducted within the 2θ range of 5–40°. Relative crystallinity (RC) was determined by dividing the crystalline integral area by the overall diffraction area, as described in reference [36].

### 3.9. FT-IR Spectroscopy Analysis

The FT-IR spectroscopy of RRP was determined by an FT-IR Spectrometer (Nicolet iS20, Thermo Fisher Scientific, Waltham, MA, USA) over a wavelength range of 400–4000 cm^−1^ [29].

### 3.10. Measurement of Total Phenolic Content (TPC)

Briefly, the sample was mixed with acidified ethanol solution (ethanol:water:HCl = 79.9:20:0.1, *v*/*v*/*v*) at a sample-to-solvent ratio of 1:20 (*w*/*v*). The mixture was magnetically stirred for 30 min and then centrifuged at 4800 rpm for 5 min. The extraction procedure was repeated four times. The supernatants from each extraction were combined, concentrated by rotary evaporation at 40 °C until nearly dry, and then reconstituted with the extraction solvent to a final volume of 10 mL. TPC was determined using the Folin–Ciocalteu method as described by Guo et al. [35]. Absorbance values were recorded at 760 nm, and TPC was quantified based on a gallic acid standard curve. The results were calculated as a gallic acid equivalent (GAE) in mg/g of RRP DW (dry weight).

### 3.11. WHC and OHC

RRP (1.0 g) was dispersed into a 20 mL plastic centrifuge tube, and 10 mL of distilled water or soybean oil was added. The mixture was fully blended and left to stand for 6 h, then centrifuged at 4000 rpm for 15 min. After removing the supernatant distilled water or soybean oil, the residue mass was weighed. The calculation formula for WHC or OHC is as follows.

*WHC or OHC* (*g*/*g*) = (*M*2 − *M*1)/*M*1

Here, M1 represents the weight of RRP, and M2 represents the weight of the precipitate after centrifugation [23].

### 3.12. Pasting Properties

A rapid viscosity analyzer (RVA, TecMaster, Perten Instruments, Warriewood, Australia) was applied to characterize the pasting behaviors of RRP suspensions, referring to the previous method [25]. Here, 3.0 g of RRP was loaded into the RVA canister, then mixed with 25.0 mL of distilled water. The mixture was kept at 50 °C for 1 min, subsequently heated to 95 °C at 14 °C/min and maintained at 95 °C for 2.5 min. Afterwards, the gelatinized sample was cooled down to 50 °C at the same constant rate and held at 50 °C for 2 min.

### 3.13. In Vitro Digestion of RRP

The in vitro starch digestibility of RRP was investigated according to a previous study [37]. α-amylase (30 mg, 13 U/mg, A3176, Sigma-Aldrich) was stirred with 10 mL of sodium acetate buffer (0.2 mol/L, pH 5.2) for 30 min. After centrifugation (4000 rpm, 10 min), the supernatant and amyloglucosidase (1 mL, 260 U/mL, A7095, Sigma-Aldrich) were mixed to form the mixed enzyme solution. The RRP (200 mg) and 5 mL water were maintained for 30 min in the boiling water bath with continuous shaking (180 rpm), followed by the addition of 15 mL of sodium acetate buffer (0.2 mol/L, pH 5.2). The mixture was maintained at 37 °C for another 10 min before adding 0.1 mL of mixed enzyme solution. Aliquots of 0.2 mL were taken out at 0, 20, 40, 60, 90, 120 and 180 min into absolute ethanol (0.8 mL) to terminate the enzymatic hydrolysis, and then the mixture was centrifuged (4000 rpm, 5 min). Subsequently, a D-glucose assay kit was utilized to measure the glucose concentration in the supernatant. Briefly, 0.1 mL of the supernatant was mixed with 3 mL of GOPOD reagent and incubated in a water bath at 50 °C for 20 min. The absorbance of the reaction mixture was then measured at 510 nm.

The concentration of glucose can be calculated as follows, according to the D-glucose assay kit (GOPOD) instruction manual:

*c* (*g*/*L*) = Δ*A_Sample_* × 100/Δ*A_Standard_* × 10 × 1/1000 × 1000/1000 × *Dilution* = Δ*A_Sample_* × 1/Δ*A_Standard_* × *Dilution*           

Here, c is the glucose concentration, Δ*A_Sample_* represents the absorbance of the sample read against the reagent blank, 100 is 100 μg of D-glucose used as standard, Δ*A_Standard_* represents the absorbance of 100 μg of D-glucose standard read against the reagent blank, 10 is the conversion from 0.1 mL of sample assayed to 1 mL, 1/1000 is the conversion from μg to mg, 1000/1000 is the conversion from mg/mL to g/L, and Dilution indicates further dilution prior to incubation with GOPOD Reagent (if required).

The calculation of starch hydrolysis was undertaken in accordance with the following equation.

*Starch Hydrolysis* (%) = *SH*/*SI* = (0.9 × *GH*)/*SI* × 100%

Here, SH stands for the hydrolyzed starch amount, SI represents the initial starch amount, and GH indicates the produced glucose amount. Further, 0.9 is a factor used to convert the molar mass from glucose into anhydroglucose (the starch monomer unit).

The total starch content was determined using a Total Starch Assay Kit. Briefly, accurately weighed duplicate samples (~100 mg) were placed in two tubes, one as the sample and the other as the sample blank, and each was mixed with 10 mL of sodium acetate buffer (100 mM, pH 5.0, containing 5 mM CaCl_2_). Thermostable α-amylase (0.1 mL, 300 U) was added to the sample tube, while buffer was added to the sample blank, followed by incubation at 100 °C for 15 min. After cooling to 50 °C for 5 min, amyloglucosidase (0.1 mL, 330 U) was added to the sample and buffer was added again to the blank, followed by incubation at 50 °C for 30 min. The mixtures were centrifuged at 13,000 rpm for 5 min. A D-glucose assay kit was utilized to measure the glucose concentration in the supernatant. The total starch content was calculated as follows:

*Starch* (%) = Δ*A* × *F* × (*EV*/0.1) × (100/*W*) × (162/180) × *D* × (1/1000)

Here, Δ*A* is the absorbance of sample solution read against reagent blank less the absorbance of the sample blank read against the reagent blank, F is the factor used to convert absorbance values to μg glucose, EV is the sample extraction volume, 0.1 is the volume of sample analyzed, D is the further dilution of sample solution, 100/W is the conversion to 100 mg sample, 162/180 is the factor used to convert from free glucose, and 1/1000 is the conversion from μg to mg. The initial starch amount was calculated by multiplying the weight of the RRP sample taken for the digestion study (~200 mg) by the total starch content (%), determined as described above.

### 3.14. Statistical Analysis

All samples were measured in triplicate (analytical replicates). Statistical analysis was conducted via SPSS 26.0 software for Windows (SPSS Inc., Chicago, IL, USA). The experimental results were expressed as mean ± standard deviation, and are reported descriptively. The graphical plotting of datasets was accomplished with Origin 2024 software.

## 4. Conclusions

The results obtained in this study show that superfine grinding induced structural modifications in RRP, thereby altering its physicochemical properties. Mechanical forces during grinding efficiently disrupted the dense tissue structure of rice bran, greatly reducing particle size and increasing specific surface area. These changes further decreased powder flowability, facilitated the release of endogenous phenolic compounds, and improved the water- and oil-holding capacities of red rice powder. Structural characterization confirmed that superfine grinding reduced starch crystallinity without altering the basic chemical characteristics of components or generating new functional groups. Moreover, the structural modifications induced by superfine grinding modulated the pasting properties and enhanced the in vitro starch digestibility of red rice powder. Different grinding gaps produced RRP with a distinct processing adaptability. The findings of this study provide fundamental data to support further research and the application of superfine ground red rice powder in diverse industrial sectors, such as functional beverages, bakery products, and gluten-free foods.

## Figures and Tables

**Table 1:** Effects of superfine grinding on particle sizes of RRP.

Samples	D(10) (μm)	D(50) (μm)	D(90) (μm)	Specific Surface Area (m^2^/kg)
R0	5.0 ± 0.1 ^c^	35.6 ± 1.1 ^a^	196.1 ± 8.3 ^a^	179.9 ± 2.8 ^c^
R1	6.2 ± 0.1 ^a^	23.8 ± 0.2 ^b^	63.6 ± 1.1 ^b^	173.7 ± 2.0 ^d^
R2	5.5 ± 0.0 ^b^	19.1 ± 0.0 ^c^	48.3 ± 0.3 ^c^	200.2 ± 0.9 ^b^
R3	4.3 ± 0.0 ^d^	14.0 ± 0.0 ^d^	32.3 ± 0.6 ^d^	255.8 ± 0.8 ^a^

The reported results correspond to mean ± standard deviation. Different letters within the same column are used to group samples with numerically different values.

**Table 2:** Color parameters of RRP with different particle sizes.

Samples	L*	a*	b*
R0	50.41 ± 0.84 ^d^	6.31 ± 0.13 ^a^	10.30 ± 0.24 ^a^
R1	56.25 ± 0.48 ^b^	4.32 ± 0.03 ^c^	7.56 ± 0.05 ^b^
R2	54.87 ± 0.20 ^c^	4.87 ± 0.10 ^b^	7.69 ± 0.19 ^b^
R3	61.11 ± 0.17 ^a^	4.11 ± 0.01 ^d^	7.53 ± 0.12 ^b^

The reported results correspond to mean ± standard deviation. Different letters within the same column are used to group samples with numerically different values.

**Table 3:** Pasting properties of RRP with different particle sizes.

Samples	PV (cP)	TV (cP)	BD (cP)	FV (cP)	SB (cP)	PT (°C)
R0	1464 ± 8 ^d^	1423 ± 8 ^b^	41 ± 1 ^c^	2996 ± 2 ^b^	1573 ± 6 ^a^	92.4 ± 0.6 ^a^
R1	2175 ± 1 ^a^	1592 ± 11 ^a^	583 ± 10 ^a^	3090 ± 2 ^a^	1498 ± 13 ^b^	86.9 ± 0.6 ^b^
R2	2043 ± 0 ^b^	1572 ± 3 ^a^	471 ± 3 ^b^	2889 ± 1 ^c^	1317 ± 1 ^c^	87.3 ± 0.0 ^b^
R3	1817 ± 13 ^c^	1374 ± 5 ^c^	444 ± 18 ^b^	2615 ± 13 ^d^	1242 ± 18 ^d^	87.6 ± 0.6 ^b^

The reported results correspond to mean ± standard deviation. Different letters within the same column are used to group samples with numerically different values. PV: peak viscosity. TV: trough viscosity. BD: breakdown. FV: final viscosity. SB: setback. PT: pasting temperature.

## Data Availability

The original contributions presented in the study are included in the article and Supplementary Materials. Further inquiries can be directed to the corresponding author.

## Supplementary Materials

The following supporting information can be downloaded at: https://www.mdpi.com/article/10.3390/molecules31203622/s1, Table S1: TPC, WHC/OHC, and digestion endpoints of RRP with different particle size.
