# Supplementary material for: Effects of Superfine Grinding on Physicochemical Properties and In Vitro Starch Digestibility of Red Rice Powder
Source: Molecules. 2026 Oct 11;31(20):3622. doi: 10.3390/molecules31203622 (PMC13707927; doi:10.3390/molecules31203622)
Supplement: Supplementary file 1 [file molecules-31-03622-s001.zip › molecules-4594819-supplementary.pdf]

Table S1. TPC, WHC/OHC, and digestion endpoints of RRP with different particle size.

Samples	TPC (mg/g DW)	WHC (g/g)	OHC (g/g)	Digestion endpoints (%)
R0	2.50±0.06^{a}	1.23±0.01^{d}	0.59±0.03^{b}	39.78±7.83^{b}
R1	2.85±0.10^{b}	1.44±0.01^{c}	0.78±0.02^{a}	80.36±0.47^{a}
R2	3.28±0.11^{c}	1.55±0.01^{b}	0.78±0.01^{a}	80.85±1.57^{a}
R3	3.20±0.25^{c}	1.61±0.03^{a}	0.77±0.01^{a}	85.44±0.14^{a}

Reported results correspond to mean ± standard deviation. Different letters within the same column are used to group samples with numerically different values.
